# The strategic role of lipidomics in biomarker identification and diagnosis of gynecological diseases

**DOI:** 10.3389/fendo.2025.1546512

**Published:** 2025-09-17

**Authors:** Menghui Hou, Xinying Chu, Shuxin Wang, Qingyue Zhang, Mingjie Ma, Jing Ma

**Affiliations:** First Teaching Hospital of Tianjin University of Traditional Chinese Medicine, National Clinical Research Center for Chinese Medicine Acupuncture and Moxibustion, Tianjin, China

**Keywords:** lipidomics, gynecological diseases, diagnosis, mass spectrometry, treatment, cell-cell communication

## Abstract

Lipidomics, an emerging field in medical research, has deepened our understanding of lipid metabolism, signal transduction pathways, and intercellular communication through qualitative and quantitative analyses of patient lipid profiles. It has closely linked these biological processes to the occurrence and progression of diseases, opening new avenues of research and providing new perspectives on the diagnosis, treatment, and personalized medicine of clinical diseases. Gynecological diseases have a profound impact on women’s health but often face challenges due to delayed diagnosis and inadequate treatment options. Lipids play a crucial role in regulating cell proliferation, differentiation, and signal transduction, making them significant in the occurrence and development of gynecological diseases. The technological progress in lipidomics has greatly advanced our comprehension of lipid metabolism and biochemical mechanisms in these diseases, while also offering new technical pathways for identifying potential biomarkers. Thus, this review summarized the application of lipidomics in gynecological diseases, especially those with high incidence rates such as ovarian cancer, cervical cancer, and endometriosis, to assesses its application potential in the diagnosis, prognosis monitoring, and development of new treatment strategies for gynecological diseases, and discusses its future development trends.

## 1 Introduction

**Figure 1:**
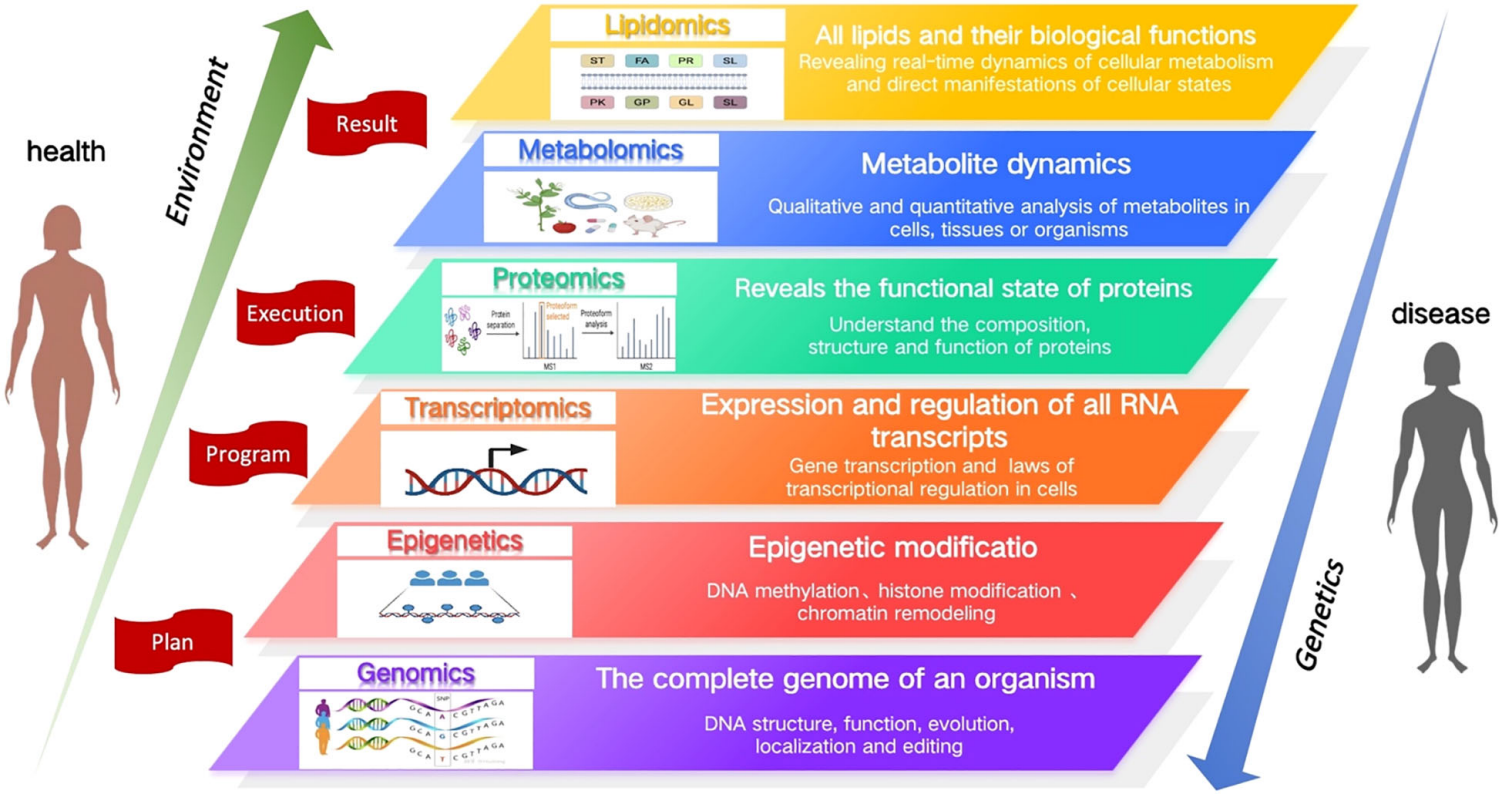
An introduction to multi-omics functions.

Omics technologies have been widely applied in the field of life sciences to systematically investigate the structure and function of organisms, aiming to uncover regulatory mechanisms. These technologies play a pivotal role in disease diagnosis, treatment, and prognosis, and are considered one of the important technical tools for advancing precision medicine. Among them, genomics serves as the foundation of omics research, providing genetic information of an organism; epigenomics investigates the regulatory mechanisms that control gene expression through chemical modifications such as DNA methylation and histone modifications; transcriptomics focuses on gene expression patterns and transcriptional regulation, revealing dynamic changes in gene expression; proteomics explores the structure and functional states of proteins, the final products of gene expression; and metabolomics analyzes small molecule metabolites and their dynamic changes within the organism. Lipidomics, focusing specifically on lipids, investigates their diverse structures, functions, and dynamic alterations. Lipids, as crucial components of cellular metabolism, reflect the ultimate effects of gene expression and protein activity under various physiological and pathological conditions. These omics disciplines complement and support each other, collectively contributing to biomarker screening, disease mechanism research, drug target identification, disease stratification, and the provision of personalized treatment. **Figure 1** summarizes the functions of each omics field.

Lipid metabolism, constituting approximately 70% of the metabolites in plasma, represents a fundamental component of the human metabolic network. Lipids, as major components of cell membranes and lipid droplets, are deeply involved in critical biological processes such as signal transduction, energy supply, and intercellular communication (1). Under pathological conditions, dysregulation of lipid metabolism is closely linked to a variety of diseases, including cardiovascular diseases, metabolic syndrome, and cancer. Lipidomics focuses on the study of these metabolic end products, revealing real-time dynamic changes in cellular metabolism, and provides a more direct reflection of biochemical alterations in disease states compared to other omics approaches (2). By bridging gene expression, protein function, and downstream metabolic outcomes, lipidomics occupies a central role in integrative multi-omics research.

Lipids play a crucial multidimensional role in the pathophysiology of gynecological diseases, influencing cellular function, signal transduction, energy metabolism, and inflammatory responses (3). For instance, in gynecological cancers, lipid metabolism is reprogrammed to support the energy demands of rapidly proliferating cancer cells (4–6). However, the molecular underpinnings of many gynecological diseases remain insufficient, with many disease etiologies yet to be fully elucidated. Diseases such as ovarian cancer and endometriosis often face diagnostic delays, while current treatment options for endometrial cancer and polycystic ovary syndrome are still inadequate to meet clinical needs. These diseases pose significant threats to women’s health, yet the existing clinical strategies remain challenging. Lipidomics, empowered by advancements in high-resolution mass spectrometry and liquid chromatography, has emerged as a powerful tool for revealing metabolic states and physiological abnormalities (7). These advancements have greatly expanded the scope and depth of lipidomics research, offering new insights into the mechanisms of gynecological diseases and laying the foundation for the development of novel diagnostic tools, personalized therapeutic strategies, and prognostic monitoring approaches. This review summarizes the latest advancements and potential applications of lipidomics in the study of gynecological diseases.

## 2 Lipidomics strategy and process: an overview

### 2.1 Lipid classification and function

**Figure 2:**
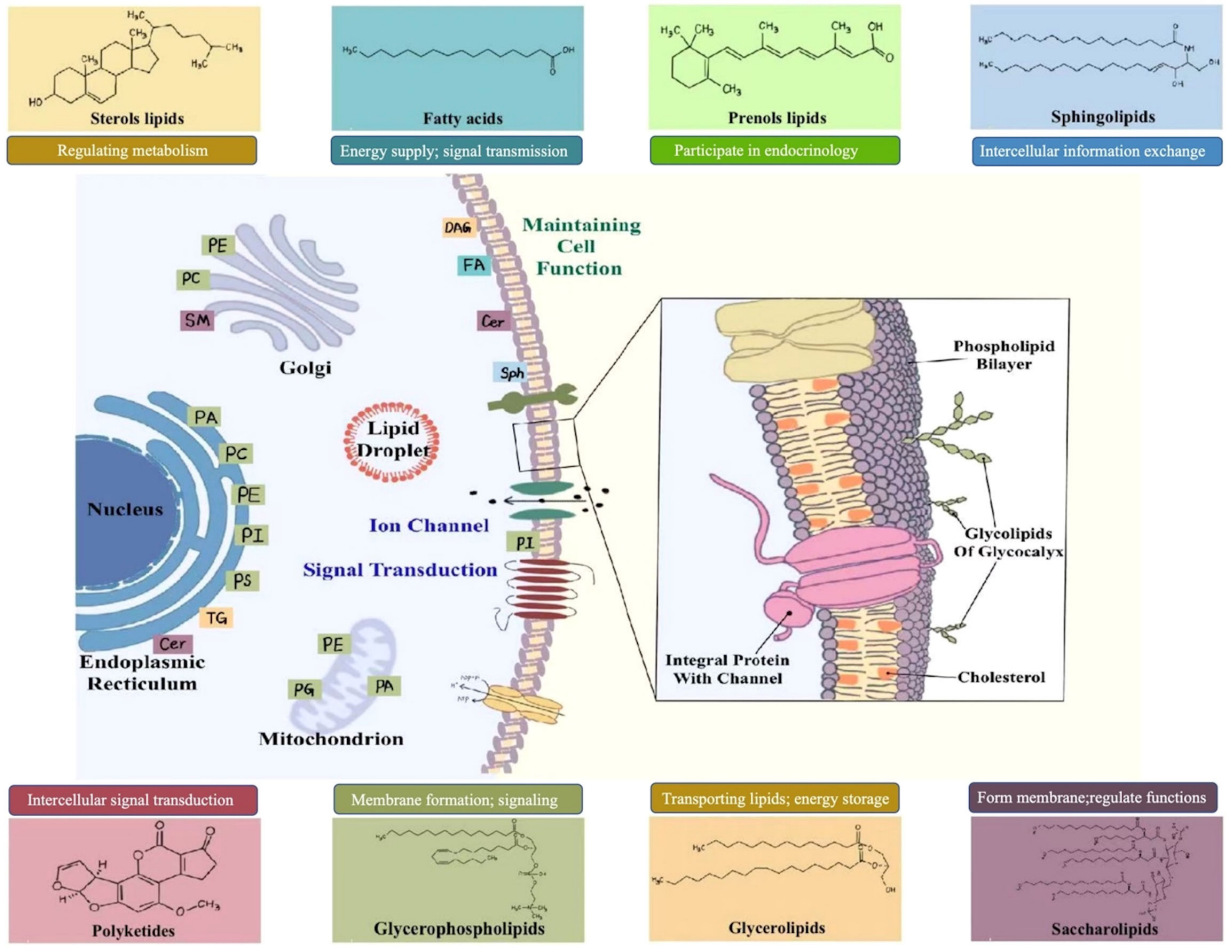
Lipid classification and function.

Lipids are part of biological membranes and are involved in the regulation of cellular activities such as signaling, immune response, and energy storage. They play essential roles in cell proliferation, survival, death, and intercellular interactions, and are constantly changing along with physiological, pathological, and environmental changes, thereby affecting the development of various diseases such as metabolic diseases, cardiovascular diseases and tumors (8). Cellular lipids are structurally diverse, containing hundreds of thousands of different molecular lipid species. In 2005, the LIPID MAPS consortium published a classification scheme that classified individual lipid molecular species into eight categories: fatty acid (FA), glycerolipid (GL), glycerophospholipids (GP), sphingolipids (SP), sterol lipids (ST), prenol lipids (PR), saccharolipids (SL) and polyketides (PK) (9). The bio-logical functions of lipid classes are generally defined by their head groups (10). The large number of aliphatic chains in lipids, varying in length, unsaturation, double bond position, cis-trans isomerism, and branched chains, further contributes to the complexity and functional diversity of lipid species (11). The complexity of their impact on biological processes is due to their unique physical and chemical properties which play major roles in essential cellular functions (12). Furthermore, lipids contribute to a wide array of physiological and pathological processes. For example, the phospholipid (PL) bilayer, which is the basic skeleton of the cell membrane, ensuring the integrity and relative independence of the cell (13). FAs and triglycerides (TGs), the energy source for many cells, maintain basic cellular activities and functions. Many lipid molecules such as arachidonic acid and lysophospholipids (LPs) act as secondary signaling molecules. Lipidomics can clearly reveal the important role of lipids in human health and disease by identifying and quantifying alterations in cellular lipid signaling metabolism, transport, and homeostasis (14–16). **Figure 2** briefly describes the classification of lipids and their function within cells.

### 2.2 Selection of appropriate lipidomics strategies for analysis

Lipidomics is the comprehensive, systematic, qualitative, and quantitative analysis and identification of lipids in organisms, tissues, and cells. The core objective of lipidomics is to elucidate the basic structure and function of lipids. By comparing the changes in lipids and their interacting molecules across different pathophysiological states, researchers can uncover the underlying relationship between lipid metabolism and physiological and pathological processes in cells, organs, and organisms. This analytical approach shows significant potential in exploring the pathogenesis of clinical diseases, identifying potential therapeutic targets, and discovering biomarkers for early diagnosis and prognostic monitoring (17).

Lipidomics technology has evolved over the past three decades, encompassing key methodological processes including lipid extraction, separation, analysis, identification, and bioinformatic interpretation (18). High-resolution mass spectrometry (HRMS) has become the cornerstone of lipidomics research owing to its exceptional sensitivity and resolution (19). This advancement has significantly facilitated in-depth structural analysis, identification of novel lipid molecules, and quantitative measurement of lipid abundance (20), which has opened new avenues for the discovery of potential disease biomarkers and deepened our understanding of the relationship between lipid metabolism alterations and disease pathogenesis.

The selection of an appropriate analytical strategy is crucial for the successful implementation of lipidomics studies. Mass spectrometry (MS)-based lipidomics can be categorized into targeted, untargeted, and pseudo-targeted approaches, each suitable for different research contexts and emphases, enabling comprehensive lipid profiling from various perspectives. Choosing the most appropriate strategy based on specific research objectives ensures both the accuracy and the interpretive value of the lipidomics data.

#### 2.2.1 Untargeted lipidomics

Untargeted lipidomics is a comprehensive and exploratory analytical approach, which aims to provide a comprehensive and unbiased analysis of organismal lipids by identifying global changes and the abundance of lipid molecules. HRMS is the tool of choice for non-targeted lipidomic analyses because of its excellent mass resolution and accuracy, particularly for elucidating the structural composition of lipids (21). HRMS techniques include Quadrupole Time-of-Flight Mass Spectrometry (Q-TOF MS), Orbitrap MS, and Fourier transform ion cyclotron resonance MS. The accurate identification and quantification of lipids are key to untargeted lipidomics, which is particularly suitable for screening novel lipid biomarkers associated with diseases. The main data acquisition modes used in untargeted workflows are data-dependent acquisition (DDA), information-dependent acquisition (IDA), and data-independent acquisition (DIA) modes. Among these, DDA/IDA, a classical mass spectrometry data acquisition mode, has a higher sensitivity and analytical throughput, allowing for a full range of lipid biomarkers (22), providing a foundation for a comprehensive understanding of the lipid composition and metabolic pathways in biological samples.

#### 2.2.2 Targeted lipidomics

Targeted lipidomics allows the precise identification and quantification of specific lipid molecules with higher accuracy and sensitivity. This approach is often used to validate key lipid molecules or potential biomarkers that have been initially identified through non-targeted lipidomics analyses (23). Targeted lipidomic studies typically employ birdshot methods to rapidly analyze large numbers of samples and identify significantly altered lipid classes, which can be coupled with MS to determine the lipid content. The main quantitative modes of targeted lipidomics are multiple reaction monitoring (MRM) and parallel-reaction monitoring, which are the most widely used techniques (24). Ultra-performance liquid chromatography-triple quadrupole mass spectrometry (UPLC-QQQ MS) is the most commonly used technique for targeted lipidomics, with a wide linear range, high sensitivity and stability, and significant advantages in the quantification of low-abundance analytes, making it highly suitable for biomarker discovery, disease diagnostics, and therapeutic research.

#### 2.2.3 Pseudo-targeted lipidomics

Pseudo-targeted lipidomics combines the advantages of both targeted and non-targeted lipidomics to ensure the detection of a sufficient number of compounds and their quantitative accuracy (25). Based on the information from non-targeted lipidomics methods using targeted technology to achieve high coverage of lipidomics data collection, comprehensive lipid analysis can be used to screen for the highest number of compounds and discover new differential lipids or lipid classes. This technique is known for its highly sensitivity, reliability, good coverage, making is suitable for the study of metabolic characteristics in complex diseases and the discovery of potential therapeutic targets (26).

### 2.3 Lipidomics methods and procedures

**Figure 3:**
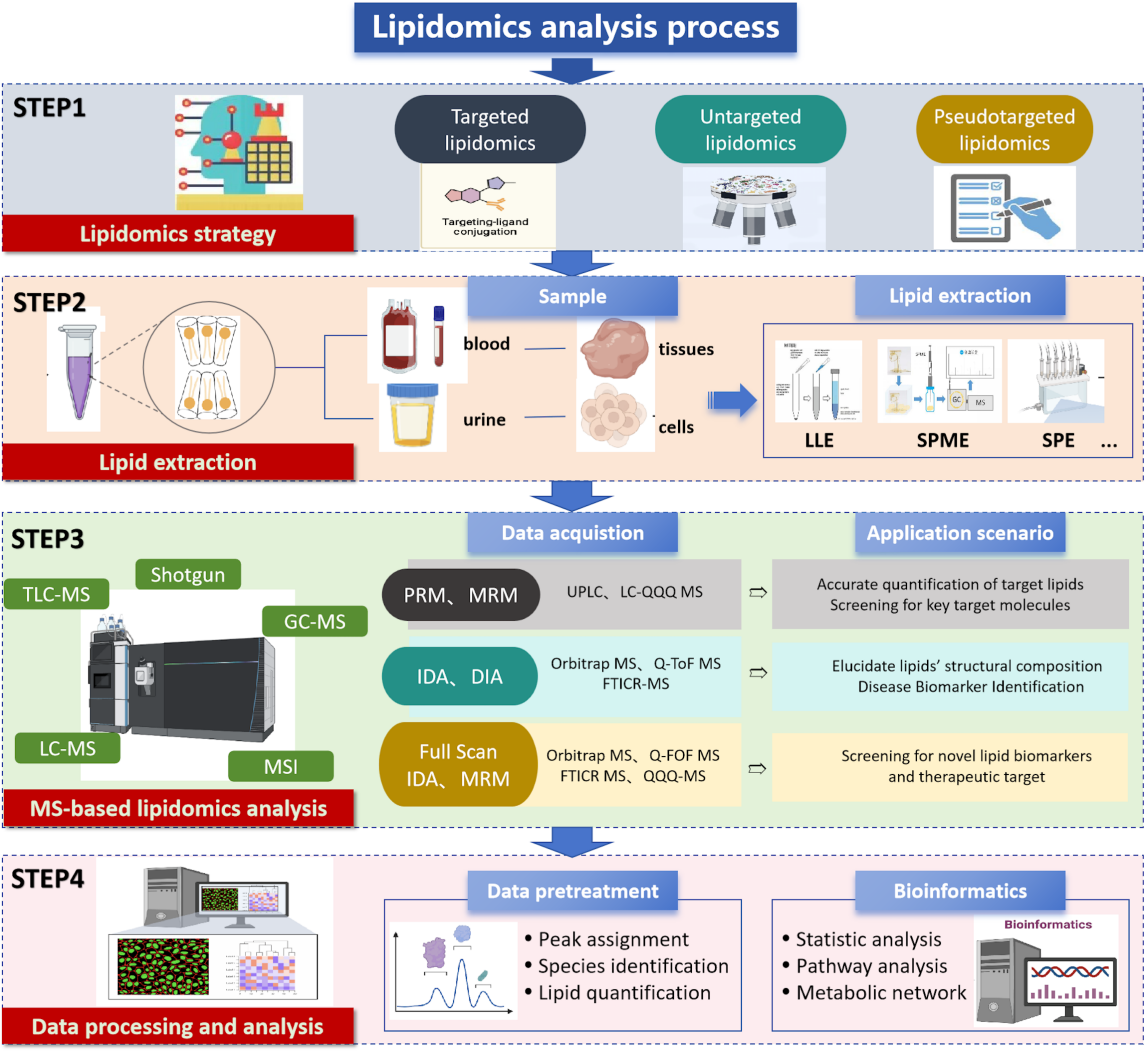
Lipidomics analysis process.

The lipidomics workflow is a complex and intricate process that encompasses the interdisciplinary intersection of chemistry, biology, computer science, and medicine (27). Each step is crucial, not only for ensuring the accuracy and reliability of experimental results, but also for deepening our understanding of lipid metabolic networks. With the emergence of new technologies, such as high-throughput sequencing, advancements in MS, and the development of bioinformatics tools, the lipidomics workflow is continuously being optimized and upgraded. The core workflow typically includes four essential steps: sample collection, lipid extraction, metabolite detection, and data analysis. These steps form the backbone of lipidomics research, and the application of new technologies infuses this framework with vitality. **Figure 3** summarizes the lipidomics analysis process.

#### 2.3.1 Sample selection

Various biological samples such as tissue, plasma, serum, urine, and exosomes can be used for lipidomic studies. To ensure the quality of the samples, they are quickly frozen in liquid nitrogen and stored at ultra-low temperatures to inhibit enzymatic activity and prevent lipid degradation. The sample should be homogenized to ensure that the extracted lipids are representative of the entire sample (28). Extraction of lipids, which follows, is central to the entire lipidomics workflow.

#### 2.3.2 Lipid extraction

Lipidomics requires efficient and reproducible sample extraction methods that cover a wide range of analytes. Commonly used extraction techniques include liquid-liquid extraction (LLE), solid-phase extraction (SPE), and solid-phase microextraction (SPME). SPE effectively enriches lipids with a very low endogenous abundance and is suitable for targeted lipidomic analysis, whereas LLE achieves comprehensive lipid extraction and is the most commonly used and well-established technique for non-targeted whole-lipid analysis (29). Folch or Bligh and Dyer method is considered to be the gold standard for lipid extraction. SPME is suitable for small sample sizes and low target concentrations. In addition, supercritical fluid extraction and ultrasonic-assisted extraction offer benefits like shorter processing times and reduced solvent consumption (30). For compounds that are difficult to detect or isolate, derivatization can change their structural properties, thereby improving the detection sensitivity, ionization efficiency, and quantitative accuracy (31, 32).

#### 2.3.3 Detection based on MS separation

Mass spectrometry-based separation and identification are central to lipidomics analysis. Lipid samples are typically first separated using chromatography techniques and subsequently identified by HRMS to determine lipid types, structural characteristics, and molecular weights. MS has become an essential tool in lipidomics research due to its high sensitivity, high resolution, and molecular specificity (33). The analytical workflow depends on the performance of the mass analyzer and the structural resolution required for lipid identification and quantification (34). Commonly chromatographic techniques include normal-phase liquid chromatography (NPLC), hydrophilic interaction liquid chromatography (HILIC), and reversed-phase liquid chromatography (RPLC), each facilitating efficient separation of different lipid species (35, 36). Sample preparation often involves ionization methods such as electrospray ionization (ESI) or matrix-assisted laser desorption ionization (MALDI) to enhance the sensitivity and detection efficiency of the analytes introduced into the mass spectrometer.

In complex lipid analysis, liquid chromatography-mass spectrometry (LC-MS) is widely employed due to its high accuracy, sensitivity and resolution, making it a primary technique for studying complex lipid mixtures. Gas chromatography-mass spectrometry (GC-MS) exhibits excellent separation capabilities, particularly for volatile compounds. Shotgun lipidomics enables high-throughput analysis through direct infusion without the need for complex separation steps, allowing for rapid generation of large-scale, high-quality data, demonstrating substantial potential for various applications (37).

During MS analysis, ionized lipid compounds are analyzed based on their mass-to-charge ratio (m/z). Quadrupole mass analyzers serve as mass filters, allowing only ions within specific m/z ranges to pass through, while time-of-flight (TOF), orbitrap, and Fourier-transform ion cyclotron resonance (FT-ICR) analyzers can acquire high-resolution spectra in a single scan. TOF analyzers determine m/z by measuring the time ions take to travel through the flight tube, while orbitrap analyzers detect image currents from trapped ions and generate spectra via Fourier transform. These high-resolution analyzers offer significant advantages for the analysis of complex samples. Tandem MS (MS/MS), which involves multiple mass analyzers, further enhances lipid molecule identification and structural elucidation. Nuclear Magnetic Resonance technology, due to its non-destructive testing advantages and efficient extraction capabilities for molecular structural information, remains an indispensable and powerful tool in lipidomics research. The selection of an appropriate analytical method based on the lipid type is essential for achieving effective detection and precise characterization.

#### 2.3.4 Data processing and raw data analysis

Following lipidomic profiling to obtain qualitative and quantitative data, rigorous data processing and analysis become imperative for discerning biologically relevant lipid species and characterizing their potential functional roles (38). The expanding adoption of mass spectrometry-based lipidomics in biomedical investigations has introduced growing complexities in data management and storage. This necessitates the implementation of sophisticated bioinformatics tools and computational platforms to effectively manage these challenges (39). Lipid databases serve as fundamental infrastructures for structural organization and data storage, functioning both as essential foundations for analytical workflows and as key knowledge bases in lipid research. Notably, LIPID MAPS and SwissLipids have emerged as the most widely employed resources. LIPID MAPS consolidates multiple specialized databases dedicated to lipid classification and structural organization (40), encompassing >59,000 lipid species with detailed annotations of molecular structures, metabolic pathways, and disease associations. These comprehensive features establish it as a critical resource for lipid identification and quantification, while facilitating data storage, retrieval, interpretation, and exploration (41, 42).

Lipidomics analyses routinely generate extensive multidimensional datasets, highlighting the critical need for appropriate software platforms to facilitate lipid identification, quantification, bioinformatics analysis, and data visualization (43). Several specialized computational tools have been developed for this purpose, including MZmine, Lipostar 2, MS-DIAL, and XCMS, which serve as essential platforms for comprehensive lipidomic data processing and interpretation. The incorporation of artificial intelligence (AI) approaches, particularly machine learning and deep learning algorithms, has significantly transformed lipidomics data analysis by enabling automated pattern recognition and feature extraction. Advances in lipidomics, particularly next-generation LC-MS/MS technologies, have significantly improved the sensitivity and accuracy of lipid analysis. The LipidSuite web server further streamlines lipidomics research by offering integrated tools for data processing, differential analysis, and functional interpretation (44). These developments, combined with AI-driven analytics, are accelerating biomarker discovery and enabling precision medicine applications in early diagnosis and targeted therapies. Ongoing innovations continue to deepen our understanding of lipid roles in disease and advance personalized treatment approaches.

## 3 The strategic role of lipidomics in the diagnosis and treatment of gynecological diseases

### 3.1 Differences in lipidomic analysis in gynecological diseases

**Figure 4:**
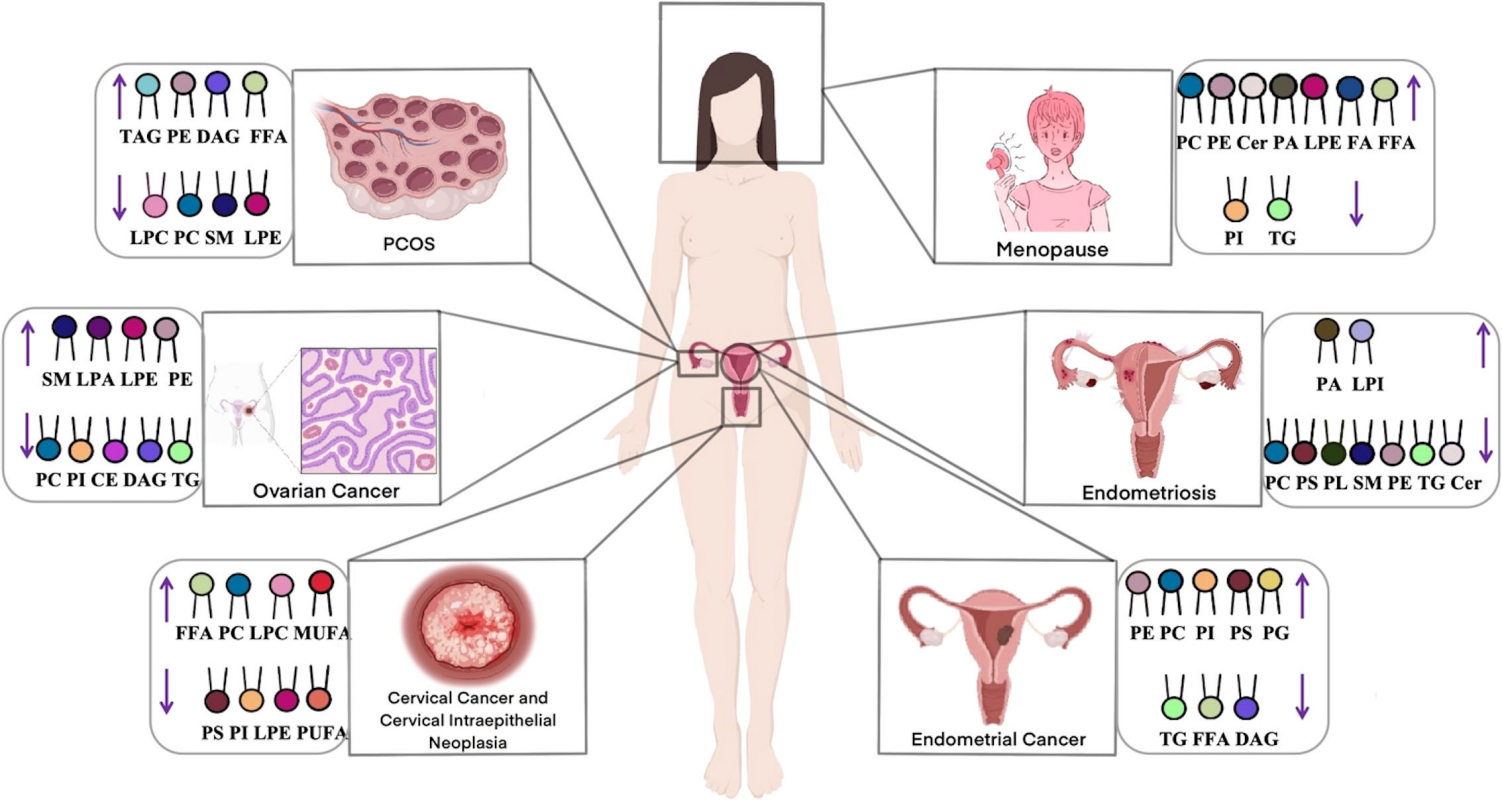
Gynecological diseases with lipid metabolism abnormalities.

Lipidomics enables comprehensive characterization of lipid profiles through qualitative and quantitative analyses, providing insights into lipid composition, signaling pathways, and their functional associations with disease states (45). This approach facilitates the investigation of disease pathogenesis, supporting early diagnosis and the development of targeted therapies. In the field of gynecology, lipidomics has been successfully applied to study ovarian cancer, cervical cancer, and endometriosis. These investigations have enhanced our understanding of how lipid metabolism dysregulation contributes to disease mechanisms while identifying potential biomarkers and therapeutic targets. In this section, we systematically review lipidomic alterations observed across major gynecological disorders. **Figure 4** summarizes the key gynecological diseases associated with lipid metabolism abnormalities.

#### 3.1.1 Ovarian cancer

Ovarian cancer, a prevalent gynecological malignancy with frequently poor prognosis due to late detection, demonstrates significant lipid dysregulation that critically influences cancer cell survival, proliferation, and invasion. Multiple lipid classes including lysophosphatidylcholines (LPCs), phosphatidylcholines (PCs), ceramides (Cers), and TGs contribute to oncogenic processes in ovarian cancer development (46), with PLs and sphingophospholipids also implicated as key players in carcinogenesis (47). Comparative lipidomic analyses reveal consistent decreases in most lipid classes (including cholesteryl esters [CEs], sphingomyelins [SMs], LPCs, and PCs) across all disease stages when compared to healthy to healthy controls (47–49), contrasting with elevated FA levels - particularly monounsaturated FAs associated with upregulated desaturases (50, 51). Notably, SMs levels have shown a positive correlation with ovarian cancer risk in postmenopausal women (52), while Cers and short-chain sphingolipids attract research interest for their apoptotic roles (53). Both targeted and untargeted approaches consistently report elevated Cers, FAs, and longer-chain triacylglycerol(TAGs) in patients (54), with UHPLC-MS/MS studies specifically documenting decreased LPC/PC alongside increased TGs levels (55). These lipid alterations functionally contribute to cancer progression, as demonstrated by Pitman’s work linking Cers, SMs, and sphingosine-1-phosphate(S1P) to proliferation, migration, angiogenesis and metastasis (48). Collectively, these findings establish glycerophospholipid metabolism as the central dysregulated pathway in ovarian cancer (46, 56), offering promising avenues for early diagnostics. The distinct lipidomic signature of ovarian cancer underscores the profound interconnection between lipid metabolism and disease pathogenesis.

#### 3.1.2 Cervical cancer and cervical intraepithelial neoplasia

Cervical cancer, a prevalent malignancy in women, frequently arises from CIN, with high-risk human papillomavirus (HPV) infection playing a pivotal role in its pathogenesis. Emerging evidence highlights the significance of lipid metabolism dysregulation in cervical carcinogenesis (57). Disease-specific lipid alterations primarily involve glycerophospholipids, including PCs and PEs, which are associated with apoptosis inhibition, impaired cellular metabolism, and enhanced proliferation. Notably, low-grade squamous intraepithelial lesions (LSIL) exhibit elevated PCs and LPCs levels, whereas high-grade squamous intraepithelial lesions (HSIL) demonstrate reduced lysophosphatidylethanolamine (LPEs), PCs, and PEs content. Comparative lipidomic profiling of CIN2/3 and cervical cancer patients via UPLC-QTOF MS revealed significant differences in 31 lipid species, including PC, PE, diacylglycerols (DAGs), and FAs, relative to healthy controls and CIN1 patients (58). Cervical cancer tissues exhibited markedly lower PC and PE levels, elevated monounsaturated fatty acids (MUFAs), and persistent lipid metabolism dysregulation. Complementary untargeted histologic analysis using UHPLC-QTOF/MS further confirmed that lipid metabolic shifts critically influence carcinogenesis at the high-grade CIN stage (59). Specifically, significant reductions in PEs, LPEs, PCs, and LPCs levels were observed in cervical cancer, offering novel insights into lipid dysregulation and potential diagnostic or therapeutic targets. The distinct lipidomic profiles of cervical cancer and precancerous lesions emphasize the integral role of lipid metabolism dysregulation in disease progression, presenting opportunities for early detection and targeted intervention.

#### 3.1.3 Endometrial cancer

Abnormal lipid metabolism is a key contributing factor in the pathogenesis of endometrial cancer. Studies analyzing endometrial cancer and healthy human serum using targeted lipidomics have identified sphingolipid (SP) and glycerophospholipid (GP) metabolism as the most significantly altered pathways (60). Most GPs, GLs, and SPs levels were elevated in patients with endometrial cancer. Furthermore, Cers levels were significantly elevated, whereas FAs levels were reduced. Altadill et al. also observed that lipids, such as PEs, PCs, PIs, phosphatidylserine (PSs), and phosphatidylglycerol, are significantly upregulated in patients with endometrial cancer (61). Conversely, several lipid species were found to be downregulated, including TG (33:0), monoacylglycerol (24:0), hexacosanoic acid, diacylglycerol (36:4), monoacylglycerol (24:1), monoacylglycerol (22:0), sterol at C27H48O5, monoacylglycerol (22:4),TG(28:0) adduct, monoacylglycerol (22:2), triglyceride (24:0), capric acid (62). A previous systematic review summarized the signaling pathways that regulate lipid metabolism in endometrial cancer, mainly including mitogen-activated protein kinase, JAK kinases/signal transducer and activators of transcription, NF-kB/Notch1, and ERRα, which play a role in reprogramming lipid metabolism (63). Elevated endometrial cancer levels are also important lipid-related changes that affect several tumor-related processes such as cell membrane structure, signaling, and cell proliferation. Collectively, these findings highlight the crucial role of lipid metabolic reprogramming in endometrial cancer pathogenesis, with specific lipid species and pathways emerging as potential diagnostic markers.

#### 3.1.4 Polycystic ovary syndrome

Approximately 70% of patients with PCOS have abnormal lipid metabolism involving various metabolic pathways, such as FAs, GLs and GPs metabolism (64). UHPLC-MS/MS analyses of follicular fluid reveal significant alterations in 53 lipid species in PCOS, including elevated TAGs, DAGs, PEs, and Hexosylceramide alpha-hydroxy fatty acid-phytospingosine (HexCer-AP), alongside reduced LPCs, PCs, and SMs compared to controls (65, 66). Multiple lipidomic studies using RPLC/Q-TOF-MS and SWATH™-MS consistently demonstrate increased GLs (TGs, DAGs) and decreased PLs (PCs, LPCs, LPEs) in PCOS patients (67–69). Notably, specific lipid species including LPC (16:0), LPC (18:2), and LPE (22:5) show marked reductions, while 3-hydroxynonanoyl carnitine and eicosapentaenoic acid are significantly elevated (69). These consistent lipidomic alterations across multiple studies demonstrate a distinct dyslipidemia pattern in PCOS characterized by elevated GLs and reduced PLs, suggesting profound disturbances in lipid homeostasis that may contribute to the pathophysiology of this condition.

#### 3.1.5 Endometriosis

Endometriosis has features such as invasion, implantation, and metastasis, which are similar to those of malignant tumors, making early recognition and diagnosis important (70). Lipid metabolites play an important role in endometriosis lesions (71). Chagovetset et al. analyzed the tissues of *in situ* and ectopic endometrium from 90 patients with endometriosis using ESI/LC-MS (72). The results showed that the levels of PLs, SMs, and PEs were downregulated in the tissues of the ectopic endometrium, and the differences in lipids were considerable. Quantification of lipid metabolites by UHPLC-ESI-HRMS revealed that serum levels of PCs and PSs were significantly reduced and Phosphatidic acid (PA) was elevated in patients with endometriosis (73). Targeted analyses further demonstrated elevated SMs and PCs levels are associated with impaired apoptosis and dysregulated lipid-mediated signaling in endometriosiss (74).Together, these studies demonstrate that endometriosis is associated with characteristic disturbances in PLs and SPs metabolism, with the observed lipid alterations potentially contributing to both the establishment and maintenance of ectopic lesions through effects on cellular survival pathways and local inflammatory microenvironments.

#### 3.1.6 Other gynecological diseases

Lipid levels in pregnant women with hypothyroidism differ significantly from those in healthy pregnant women and are associated with adverse pregnancy outcomes. Studies using untargeted LC-MS found that PCs and PEs levels were elevated, whereas SMs was downregulated in pregnant women with hypothyroidism compared to normal pregnant women (75), which could be a potential therapeutic target. In addition, lipidomic analysis revealed an increase in lipid species in postmenopausal women compared to premenopausal women, and elevated levels of PCs, PEs, Cers, LPCs, LPEs, and FAs were also observed (76–78), providing new understanding for the study of metabolic profiles associated with menopause.

### 3.2 Diagnostic and treatment value of lipidomics in gynecological diseases

#### 3.2.1 Potential as a diagnostic marker

**Figure 5:**
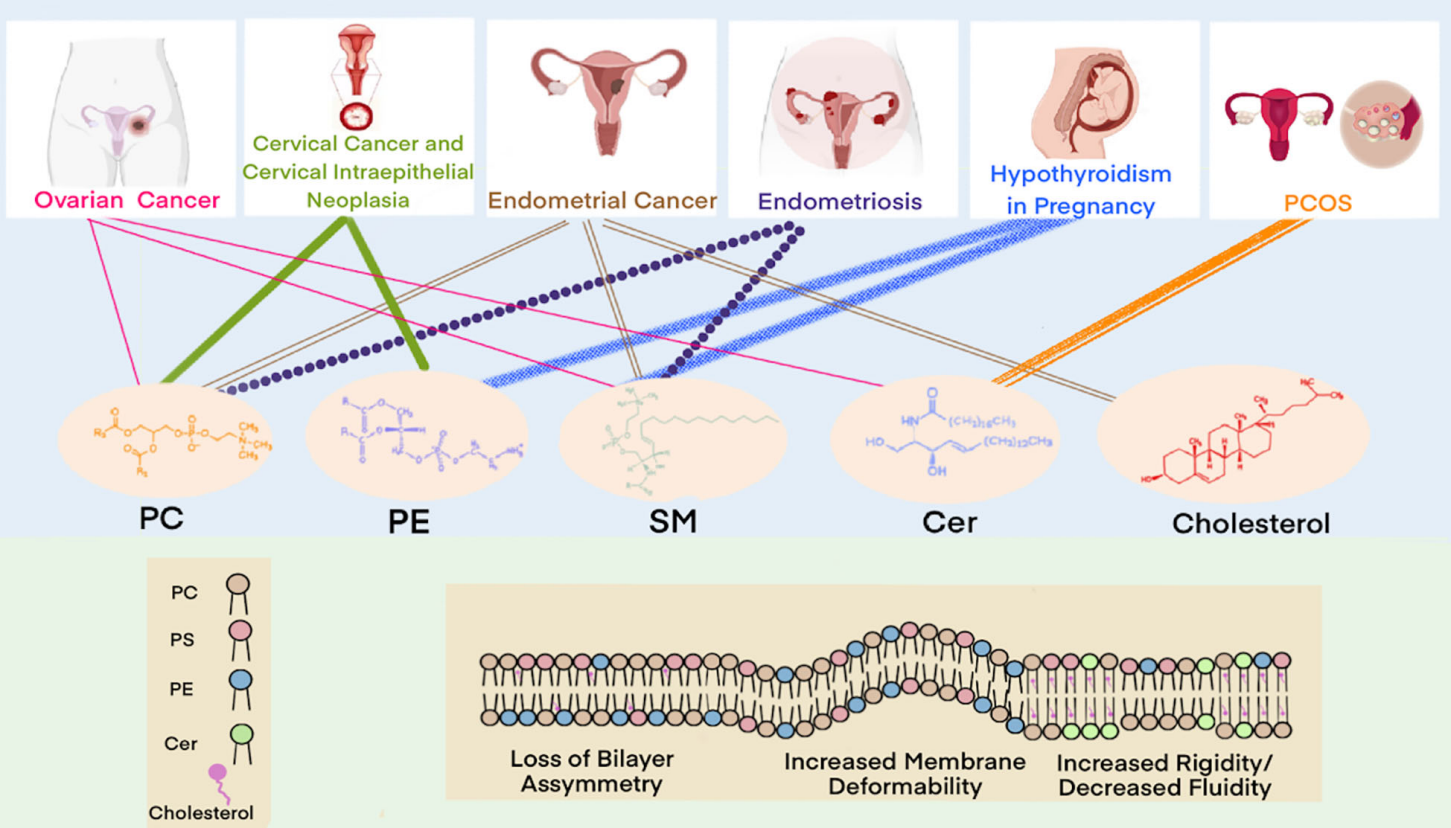
Lipids involved in diagnostic markers for gynecological diseases.

Lipidomics has emerged as a powerful approach for elucidating the pathogenesis of gynecological diseases through comprehensive qualitative and quantitative analysis of lipid profiles. These investigations provide critical insights for disease diagnosis and therapeutic development. As summarized in **Figure 5** and **Table 1**, numerous lipids show potential as diagnostic biomarkers across various gynecological conditions.

**Table 1:** Lipid biomarkers and analytical strategies for gynecological diseases.

Gynecological diseases	Lipids biomarkers	Analytical strategy	MS	References
Ovarian cancer	lysoPC a C16:1, PC aa C32:2, PC aa C34:4, and PC aa C 36:6	targeted	UHPLC-MS	(79)
	C16-Cer, C18:1-Cer, and C18-Cer	targeted	UHPLC/MS/MS	(82)
	LPA, PPE, LPC (14:0, 12:0) with CA125	targeted	ESI/LC - MS	(49)
	Cer(d18:1/16:0), PC-O(36:2), PE (16:0p_18:1) and (O-acyl)-1-hydroxy FA (18:2_24:6)	untargeted	UPLC-MS	(80)
	lysophosphatidylglycerol (20:5)	untargeted	UPLC/MS	(83)
Cervical cancer and cervical intraepithelial neoplasia	PC 14:0/18:2, PE 15:1e/22:6, and PE 16:1e/18:2	untargeted	UHPLC/Q-TOF-MS	(59)
	Cer CE (29:1), sphinganine (d18:0) and a Cer could not be firmly characterized	untargeted	HLPC/Q-TOF-MS	(94)
	prostaglandins, PLs, SFs, Tetranor-PGFM and hydroperoxide lipid	untargeted	ESI/Q-Orbitrap MS	(95)
	LPC O-16:0, PE P-16:0/20:4, and sphingomyelins SM d16:0/18:1, SM d22:0/20:3, SM d18:0/16:0	untargeted	ESI-QTOF MS	(96)
Endometrial cancer	6-keto-PGF1α, PA (37:4), LysoPC (20:1) and PS (36:0)	untargeted	UPLC-Q-TOF/MS	(97)
	acylcarnitine C16: phosphatidylcholine PCae C40:1, proline: tyrosine, PCaa C42:0 and PCae C44:5	targeted	ESI-MS	(98)
Endometriosis	hydroxyl sphingomyelin SMOH C16:1, phosphatidylcholine PC aa C36:2, and ether PLs PCae C34:2	targeted	ESI-MS/MS	(74)
	DAG, TAG, LPI, acylcarnitine	untargeted	UPLC-MS	(84)
	C0/PC ae C36:0 and PC aa C30:0/PC ae C32:2	targeted	ESI-MS/MS	(85)
	cardiolipin CL 16:0_18:0_22:5_22:6 and plasmenylethanolamine PE P-16:0/18:1	untargeted	HPLC-MS	(99)
PCOS	PI (18:0/20:3)-H and PE (18:1p/22:6)-H	untargeted	UHPLC-Q-Exactive Orbitrap MS	(86)
	Cer (OH_N16:0/N18:0) and Cer (N22:0)	untargeted	MDMS-SL	(87)

In cervical carcinogenesis, dysregulated lipid metabolism has been implicated in the progression from cervical intraepithelial neoplasia to invasive carcinoma. Rapid shotgun lipidomics of cervical tissue transformation stages revealed significant alterations in PCs, LPCs, PEs, LPEs, and SMs compared to adjacent normal tissue. ESI-MS-based analysis identified 23 signature lipids strongly correlated (more than 90%) with cervical transformation grade, forming a diagnostic model with 88% sensitivity and 71% specificity (57). Further validation through UHPLC/Q-TOF-MS untargeted analysis demonstrated that specific phospholipid combinations (PC 14:0/18:2, PE 15:1e/22:6, and PE 16:1e/18:2) could effectively discriminate between early-stage cervical cancer, squamous intraepithelial lesions, and healthy controls (59).

In ovarian cancer biology, lipid metabolism plays a crucial role, with emerging lipidomic signatures showing significant diagnostic potential. UHPLC-MS analyses have identified several novel lipid biomarkers, including lysophosphatidylcholines (LysoPC) a C16:1, PC aa C32:2, PC aa C34:4, and PC aa C36:6, which demonstrate clinical utility for ovarian cancer detection (79). Comprehensive serum lipid profiling of 153 samples via UHPLC-MS identified a diagnostic panel comprising Cer (d18:1/16:0), PC-O(36:2), PE (16:0p/18:1), and (O-acyl)-1-hydroxy FA (18:2/24:6) with high classification accuracy (80). Notably, the combination of CA125 with dysregulated phospholipids (particularly LPCs and PCs) significantly improves diagnostic sensitivity for early-stage disease (81). SPs metabolism appears particularly relevant, with Cers serving as both structural membrane components and signaling molecules. Elevated levels of specific Cers species (C16-Cer, C18:1-Cer, and C18-Cer) in ovarian cancer patients highlight their potential as mechanistic biomarkers in disease pathogenesis (82, 83).

SMs and PCs demonstrate significant associations with endometriosis pathogenesis. ESI-MS analysis identified a diagnostic model incorporating hydroxyl sphingomyelin SM (OH) C16:1, phosphatidylcholine PC aa C36:2, and ether phospholipid PC ae C34:2, which achieved 90.0% sensitivity and 84.3% specificity for detecting ovarian endometriosis (74). Complementary findings from UPLC-MS-based untargeted lipidomics of endometrial fluid revealed distinct metabolic perturbations, including decreased DAGs and TAGs alongside elevated lysophosphatidylinositols (LPIs) and acylcarnitines in endometriosis patients (84). While the predictive model from this study showed perfect specificity (100%), its sensitivity remained moderate (58.3%), suggesting potential utility in rule-in diagnostic scenarios. Further validating these findings, ESI-MS analysis of peritoneal fluid demonstrated that lipid ratio-based biomarkers (C0/PC ae C36:0 and PC aa C30:0/PC ae C32:2) could discriminate ovarian-type endometriosis with 82.8% sensitivity and 94.4% specificity (85). These collective results underscore the diagnostic potential of lipidomic profiling for endometriosis detection, particularly through minimally invasive approaches.

Recent lipidomics studies have identified several lipid species as potential biomarkers for PCOS. PI) (18:0/20:3)-H and PE (18:1p/22:6)-H were proposed as diagnostic candidates, with a biomarker panel achieving an area under the curve (AUC) of 0.815 in the test set, demonstrating 74% accuracy, 88% specificity, and 70% sensitivity (86). In another study, multi-dimensional mass spectrometry-based shotgun lipidomics (MDMS-SL) revealed Cer species Cer (OH_N16:0/N18:0) and Cer (N22:0) as novel predictive lipid markers for PCOS (87). LC-MS based studies in pregnant women with hypothyroidism revealed that elevated levels of PCs, LPCs, and PEs were significantly associated with adverse pregnancy outcomes, including preterm labor, low birth weight, and preterm rupture of membranes, suggesting their potential as prognostic biomarkers for maternal-fetal health (85). With the increasing understanding of lipids and progress in lipidomic detection methods, analytical methods, and databases, lipidomics is poised to play an increasingly important role in integrated multi-omics analyses. This integration holds great promise for enhancing molecular network mining in gynecological diseases and advancing their prediction, diagnosis, and personalized treatment strategies.

#### 3.2.2 Potential for treatment and prognosis

Lipidomics has uncovered fundamental alterations in lipid metabolism that provide novel targets for the diagnosis and treatment of gynecologic malignancies. In ovarian cancer, bioactive lipids such as Cers, sphingosine, and S1P critically regulate tumor angiogenesis and metastatic progression, offering promising avenues for both biomarker development and targeted therapy (48). Cer, in particular, functions as a pro-apoptotic mediator that enhances chemosensitivity in ovarian cancer cells (88), while lysophosphatidic acid (LPA), elevated in epithelial ovarian cancer ascites as demonstrated by LC-MS, promotes malignant behavior and chemoresistance (89). Therapeutic targeting of LPA-producing autotaxin may further improve outcomes by overcoming immunotherapy resistance (90).

In endometrial cancer, lipidomic profiling enables preoperative risk stratification to guide surgical management, while metabolic interventions such as stearoyl-CoA desaturase 1 (SCD1) inhibition suppress tumor growth (88). Additionally, statins, known modulators of lipid metabolism, have demonstrated adjuvant therapeutic potential by improving prognosis and reducing mortality in EC patients. Similarly, in cervical cancer, fatty acid supplementation has been shown to enhance radiotherapy efficacy (91). Beyond oncology, lipid-targeted strategies show broad applicability in gynecologic diseases, including the use of adipokines for managing PCOS-associated metabolic dysfunction (93) and advanced liposomal drug delivery systems to optimize therapeutic efficacy while minimizing toxicity (89).

The integration of lipidomics with multi-omics approaches is revolutionizing our understanding of gynecologic diseases, revealing not only disease-specific lipid signatures but also actionable therapeutic vulnerabilities (92, 93). Lipidomics provides a framework for precision medicine, enabling risk stratification, biomarker-driven diagnostics, and mechanism-based therapies. As lipid-centric therapies advance, their synergy with conventional and immunotherapies will be critical in shaping next-generation management strategies for gynecologic disorders.

## 4 Conclusion

In this comprehensive review, we have delineated the strategic significance of lipidomics in the diagnostic and therapeutic landscape of gynecological diseases, with a particular emphasis on its role in ovarian, cervical, and endometrial pathologies. The aberrations in lipid metabolism are not only a common pathological hallmark of these diseases but also intricately linked to disease progression. Advances in high-resolution mass spectrometry have propelled lipidomics forward, enabling detailed structural elucidation of lipids, identification of novel lipid species, and quantification of lipid abundance, thereby unlocking new avenues for the discovery of biomarkers and therapeutic targets in gynecological diseases.

Despite the substantial potential demonstrated by lipidomics in gynecological research, challenges remain in the realms of data standardization, clinical translation, and complex data analysis. Heterogeneity in sample preparation and variability in preprocessing can lead to inconsistent outcomes, while the sophisticated nature of data analysis necessitates specialized bioinformatics support, limiting its broader clinical application. Future studies should prioritize the optimization of sample processing workflows and analytical protocols to enhance precision and reproducibility of lipidomic research.

As innovations in material science, analytical instrumentation, and artificial intelligence continue to evolve, lipidomic analysis is expected to become increasingly precise, high-throughput, and clinically applicable. Lipidomics is poised to play a central role in the era of precision medicine by enabling mechanistic insights into disease pathogenesis and supporting the development of individualized therapeutic strategies. Beyond treatment, lipidomics also holds significant promise for disease prevention and early diagnosis, offering a window of opportunity for timely intervention. With the ongoing integration of lipidomics into multi-omics frameworks, a more comprehensive understanding of gynecological diseases is emerging. This systems-level approach will empower clinicians and researchers alike to identify novel biomarkers, refine risk stratification, and design mechanism-based therapies, ultimately advancing the standard of care and improving patient outcomes in gynecological medicine.
